# Supplementary material for: The Use of Infrared Thermography (IRT) in Burns Depth Assessment: A Diagnostic Accuracy Meta-Analysis
Source: Eur Burn J. 2022 Sep 1;3(3):432–46. doi: 10.3390/ebj3030038 (PMC11571867; doi:10.3390/ebj3030038)
Supplement: Supplementary file 1 [file ebj-03-00038-s001.zip › ebj-1715582-supplementary.pdf]

european burn journal
MDPI

File S1

((thermography).ti,ab OR (thermal imaging).ti,ab OR (thermograph*).ti,ab OR (thermal imag*).ti,ab OR (infrared imag*).ti,ab OR (forward-looking infrared).ti,ab OR (flir).ti,ab OR (dynamic infrared thermography).ti,ab OR (dirt).ti,ab OR (irt).ti,ab OR (infrared imaging).ti,ab) AND ((burns).ti,ab OR (burn*).ti,ab OR (burn depth).ti,ab)
